# Draft Genome Sequence of a Vancomycin-Resistant and Vancomycin-Dependent *Enterococcus faecium* Isolate

**DOI:** 10.1128/genomeA.00059-16

**Published:** 2016-04-07

**Authors:** Marion Blaschitz, Sarah Lepuschitz, Laura Wagner, Franz Allerberger, Alexander Indra, Werner Ruppitsch, Steliana Huhulescu

**Affiliations:** a Institute of Medical Microbiology and Hygiene, Austrian Agency for Health and Food Safety, Vienna, Austria; b Department of Biotechnology, University of Natural Resources and Life Sciences, Vienna, Austria

## Abstract

Vancomycin-resistant enterococci have emerged as major nosocomial pathogens worldwide. While antimicrobial pressure promotes nosocomial colonization with these enterococci, prolonged exposure to vancomycin may foster the transition from vancomycin resistance to vancomycin dependence. Here, we report the draft genome sequence of a vancomycin-dependent *Enterococcus faecium* isolate showing partial teicoplanin dependence.

## GENOME ANNOUNCEMENT

Vancomycin-resistant enterococci (VRE) are major nosocomial pathogens worldwide. While antimicrobial pressure promotes nosocomial colonization with VRE, prolonged exposure to vancomycin may foster the transition from vancomycin resistance to dependence (1). Enterococci showing growth on medium containing 6 µg/ml vancomycin and an MIC of >8 µg/ml are considered vancomycin resistant. Strains unable to grow in the absence of 6 µg/ml vancomycin, despite multiple subcultures, are considered vancomycin dependent (1). Vancomycin-dependent enterococcus (VDE) was first described in 1993 (2, 3). To our knowledge, only twenty-five cases of VDE have been described worldwide so far (4).

We report here the draft genome of a VRE/VDE isolate obtained in Austria in 2007 from an oncology patient 1 month after cessation of teicoplanin therapy (5). The described strain of VDE showed resistance to penicillin (MIC, 64 µg/ml), ampicillin (MIC, 128 µg/ml), amoxicillin-clavulanate (amoxicillin MIC, >256 µg/ml), erythromycin (MIC, 8 µg/ml), clindamycin (MIC, 8 µg/ml), ciprofloxacin (MIC, >2 µg/ml), moxifloxacin (MIC, >2 µg/ml), fusidic acid (MIC, 4 µg/ml), and low-level resistance to gentamicin (MIC, 32 µg/ml) when tested according to Clinical and Laboratory Standards Institute (CLSI) standards (6). The strain was susceptible to quinupristin-dalfopristin (MIC, <0.5 µg/ml), oxytetracycline (MIC, <0.5 µg/ml), linezolid (MIC, 2 µg/ml), chloramphenicol (MIC, 8 µg/ml), and mupirocin (MIC, 2 µg/ml). The strain showed intermediate resistance to teicoplanin when tested on Mueller-Hinton agar (bioMérieux, Marcy l’Etoile, France) using a 30-µg teicoplanin disc (Oxoid, Basingstoke, United Kingdom) or a teicoplanin Epsilon test strip (AB Biodisk, Solna, Sweden) (MIC, 8 µg/ml). It also showed partial teicoplanin dependence, i.e., it grew in the area of low concentration of teicoplanin (0.016 to 6 µg/ml) but was inhibited in the area with high teicoplanin concentrations (8 to 256 µg/ml). The strain showed high-level resistance to vancomycin (MIC, >256 µg/ml) and complete vancomycin dependence when tested on Mueller-Hinton agar (bioMérieux) with a 30-µg vancomycin disk (Oxoid) or a vancomycin Epsilon test strip (AB Biodisk). The strain also showed *in vitro* reversion to vancomycin-nondependent vancomycin-resistant *Enterococcus faecium* mutants.

The MagAttract high-molecular-weight (HMW) DNA kit (Qiagen, Hilden, Germany) was used to isolate genomic DNA from overnight cultures grown on Mueller-Hinton agar plates (bioMérieux) with a 30-µg vancomycin disk (Oxoid) and a 30-µg teicoplanin disk (Oxoid). The fragment library was prepared using the Nextera XT kit (Illumina, Inc., San Diego, CA) and 1 ng of genomic DNA. Paired-end sequencing (2 × 300 bp) was performed on a MiSeq (Illumina, Inc.), generating 1,365,744 reads from 333,173,006 unassembled nucleotides. Raw reads were *de novo* assembled into a draft genome using Velvet version 1.1.04 (7). Contigs were filtered for a minimum coverage of 5 and minimum length of 200 bp, which resulted in 232 contigs with a total of 2,949,766 nucleotides at a coverage of 95-fold. A total of 2,953 genes, 2,878 coding sequences, 95 pseudogenes, 7 rRNA genes, and 64 tRNA genes were identified using the NCBI Prokaryotic Genome Annotation Pipeline (http://www.ncbi.nlm.nih.gov/genome/annotation_prok/).

### Nucleotide sequence accession numbers.

This whole-genome shotgun project has been deposited at DDBJ/EMBL/GenBank under the accession no. LQRS00000000. The version described in this paper is the first version, LQRS01000000.
